# The Hospital World

**Published:** 1910-12-17

**Authors:** 


### The Hospital World.

### THE LATE MR. WILLIAM JOHN NIXON.

On December 7 there died at Brighton, at a ripe old age, a gentleman who for many years had been regarded as the *doyen* and leader of hospital officials. We refer to Mr. W. J. Nixon, a former Secretary of the London Hospital, who died after a week's illness from pneumonia. Mr. Nixon was appointed to this post in 1846, becoming House Governor in 1866. He retired in 1892. Everybody who knew him describes him as a man of great nobility of character, enormous energy, of unswerving justice, and a strict disciplinarian.

Mr. Nixon was a man who was intensely interested in his work as House Governor of the London Hospital, very particular as to details, precise and exact as to punctuality. He was very keen indeed on statistics, and was continually working out averages and balancing those of his own hospital against the information he was able to acquire from other institutions. All this work is now done elaborately by the Central Committees, such as the King's Fund.

The particular value of the statistics as worked out by Mr. Nixon was that he, as an expert, made inquiries, fixing his attention definitely on certain objects in regard to which he desired to obtain strictly accurate information. He was, therefore, able to make his comparisons a particularly valuable guide to his committee.

An old private secretary of Sir Thomas Fowell Buxton, Mr. Nixon first went to the London Hospital and was there through the distinguished chairmanships of Thomas Fowell Buxton, O. E. Coope, Sir Edmund Currie, and John Henry Buxton, all of them men who devoted an immense amount of time and ability to furthering the interests of the East-End charity with which they were so closely identified.

Mr. Nixon often spoke of the days when extra nurses were hired for the day's work from among those applicants who came and sat on the railings outside waiting for their turn to be employed. He lived to see the thorough establishment of trained nurses at the London Hospital under the able management of the present matron, Miss Luckes. He was very strict in looking after the house officers' attendance on their duties, and was himself so punctual in all matters that he made very little variation in the routine of his day's work.

Mr. Nixon's great relaxation was mountaineering, and until the very last years of his career at the London Hospital he never missed a mountaineering holiday in Switzerland. The blindness with which he was afflicted in the latter days of his life was a very sore trial to him, and completely incapacitated him. He was pensioned by the hospital in recognition of the valued work he had done.

We have received the following appreciation by an old friend:—

Mr. Nixon was born at Norwich, February 15, 1821, his father being a solicitor there, and was educated at the Grammar School, being head boy when he left, and having as second boy the late J. S. Perowne, who eventually became Bishop of Worcester. Mr. Nixon became a good classical scholar, the advantages of which he felt throughout his life, and we may mention here that his official letters were models of clearness, of sound, terse English, and of good composition.

While still young (probably under eighteen), he became private secretary to the first Sir Thomas Fowell Buxton, Bart., and proved for many years the latter's "right hand" in his Parliamentary labours for the suppression of the Slave Trade. Sir Fowell Buxton must have been a man of the finest qualities of heart and mind, and to serve under such a man must have been in itself a liberal education to the youth, who always spoke of him with the highest admiration and affection.

A few months after Sir Fowell's death there occurred a vacancy in the secretaryship of the London Hospital, and Mr. Nixon was put forward as a candidate. He was only two months on the right side of the minimum age (twenty-five) for the appointment, but the impression made by himself and by the striking testimonials that supported him carried the day, and from 1846 to 1892 his life was devoted to the service of the London Hospital.

In 1866, on the retirement of Mr. Robert J. Hill, Mr. Nixon became House Governor as well as Secretary, only giving up the latter post when, nine years later, the duties of the combined offices had become too great for one man to undertake.

It may be mentioned that for many years he had acted as Secretary to the Gas Company of the district in which he resided—at that time the duties would by no means be exacting—and this appointment he relinquished upon going to live at the Hospital as House Governor.

Mr. Nixon was a man of large and liberal mind, of unusual power of application and mental concentration, and of the utmost personal probity and honour. His heart and thought were always devoted to his hospital, and it is not too much to say that it was mainly owing to his work and personal character that the London Hospital enjoyed that public confidence which was always shown it, and made it—what under him it undoubtedly became—the foremost charity of its kind in England.

His health broke down in 1892, and he was compelled to retire from active life. The Hospital of September 24, 1892, page 431, contained a full report of the proceedings at the Special General Court of the Governors of the London Hospital at which Mr. Nixon's resignation was accepted, and of the high testimony that was borne on that occasion to his abilities. A handsome retiring allowance was at the same time unanimously voted to him. The dreadful calamity of total blindness—caused by gout—befell him, unfortunately, very shortly after his retirement.

To a man of his active habits this was, of course, a terrible trial, but few people probably bore it more patiently. His last eighteen years were mostly spent at Brighton.

Mr. Nixon was a well-read and well-informed man, books of travel being, perhaps, his favourite reading. In the course of his long life he had naturally made the acquaintance of many people of mark, and his reminiscences could be most entertaining.

In the early days of the Volunteer movement Mr. Nixon joined the 5th Essex, and was known as a capable captain and one of the best shots of the corps.

He delighted in fine scenery, and was very familiar with the Lake District of England and the mountains of Scotland. His favourite "hunting-ground," however, was Switzerland, where he had spent some thirty-three summer holidays, climbing or crossing most of the chief mountains and passes.

A man of slim build (about 5 feet 7 inches in height), his power of "lasting" was wonderful, and would often astonish even the guides who knew him best. With these fellows, as also with the old-time landlords of the hotels at which he stopped from year to year, Mr. Nixon was a great favourite, for his charm of manner, no less than his sterling character, attracted all who came in contact with him.

